# EXAMINING SPIRITUALITY, COGNITIVE, AND FUNCTIONAL HEALTH IN OLDER U.S. ADULTS

**DOI:** 10.1093/geroni/igae098.4175

**Published:** 2024-12-31

**Authors:** Katherine Britt, Fanghong Dong, Lauren Massimo, Jill Hamilton, Nancy Hodgson, Shana Stites, Dawn Mechanic-Hamilton

**Affiliations:** The University of Pennsylvania, Philadelphia, Pennsylvania, United States; Washington University at St. Louis, St. Louis, Missouri, United States; The University of Pennsylvania, Philadelphia, Pennsylvania, United States; Emory University, Atlanta, Georgia, United States; The University of Pennsylvania, Philadelphia, Pennsylvania, United States; The University of Pennsylvania, Philadelphia, Pennsylvania, United States; The University of Pennsylvania, Philadelphia, Pennsylvania, United States

## Abstract

Used as a salient resource among Black communities to cope with stress, spirituality and religion are associated with better overall cognitive function. However, their impact on individual cognitive domains and functional health is limited, and understanding these relationships could inform targeted interventions for optimizing cognitive and functional health. Using data from cognitively unimpaired older Black and White U.S. adults in 2021-2022 from the Aging Brain Cohort (ABC) at The University of Pennsylvania Alzheimer’s Disease Research Center, we examined cross-sectional associations between spirituality (measured with one item, “To what extent do you consider yourself a spiritual person?”), religiousness (measured with one item, “To what extent do you consider yourself a religious person?”), cognition (i.e., memory measured with Benson Delayed Recall and executive function measured with Trail Making B) and functional health (measured with the Functional Rating Scale (FRS) and Functional Activities Questionnaire (FAQ) (N=158) using multivariable regression analysis. Participants were 27.8% Black, 64.6% women, with mean age (74.4 years) and education (16.7 years). Controlling for age, education, sex, and social interaction, spirituality was positively associated with memory (β = 0.19, 95%CI [0.05, 0.33], p<.007) but not executive function or functional health. In comparison, religiousness was positively associated with functional health (FAQ only)(β = 0.31, 95%CI [0.01, 0.61], p<.046)) but not memory or executive function. Older Black and White adults with greater spirituality had higher memory scores, and those with greater religiousness had a higher functional health score. Longitudinal studies are needed to examine associations further.

